# Health Tract, No. 13

**Published:** 1865

**Authors:** 


HEALTH TRACT, NO. 13.

### SITTING POSITION.

### THE TRUE PHYSIOLOGICAL CHAIR.

ALL consumptive people, and all afflicted with spinal deformities, sit habitually crooked, in one or more curves of the body. There was a time in all these when the body had its natural erectness, when there was not the first departure on the road to death. The make of our chairs, especially that great barbarism, the unwieldy and disease-engendering rocking-chair, favors these diseases, and undoubtedly, in some instances, leads to bodily habits from which originate the ailments just named, to say nothing of piles, fistula, and the like. The painful or sore feeling which many are troubled with incessantly for years, at the extremity of the back-bone, is the result of sitting in such a position that it rests upon the seat of the chair, at a point several inches forward of the chair-back. A *Physiological chair*, one which shall promote the health, and preserve the human form erect and manly as our Maker made it, should have the back straight, at right angles with the seat, the seat itself not being over eight inches deep. A chair of this kind will do more toward correcting the lounging habits of our youth, than multitudes of parental lecturings, for then if they are seated at all, they must sit erect, otherwise there is no seat-hold.

A very common position in sitting, especially among men, is with the shoulders against the chair-back, with a space of several inches between the chair-back and the lower portion of the spine, giving the body the shape of a half hoop; it is the instantaneous, instinctive, and almost universal position assumed by any consumptive on sitting down, unless counteracted by an effort of the will; hence parents should regard such a position in their children with apprehension, and should rectify it at once.

